# Downregulation of autophagy is associated with severe ischemia-reperfusion-induced acute kidney injury in overexpressing C-reactive protein mice

**DOI:** 10.1371/journal.pone.0181848

**Published:** 2017-09-08

**Authors:** Ao Bian, Mingjun Shi, Brianna Flores, Nancy Gillings, Peng Li, Shirley Xiao Yan, Beth Levine, Changying Xing, Ming Chang Hu

**Affiliations:** 1 Department of Nephrology, the First Affiliated Hospital of Nanjing Medical University, Nanjing, Jiangsu, China; 2 Charles and Jane Pak Center for Mineral Metabolism and Clinical Research, University of Texas Southwestern Medical Center, Dallas, TX, United States of America; 3 Departments of Pathology, University of Texas Southwestern Medical Center, Dallas, TX, United States of America; 4 Departments of Internal Medicine, University of Texas Southwestern Medical Center, Dallas, TX, United States of America; 5 Departments of Microbiology, University of Texas Southwestern Medical Center, Dallas, TX, United States of America; 6 Center for Autophagy Research, University of Texas Southwestern Medical Center, Dallas, TX, United States of America; 7 Howard Hughes Medical Institute, University of Texas Southwestern Medical Center, Dallas, TX, United States of America; Hopital Tenon, FRANCE

## Abstract

C-reactive protein (CRP), was recently reported to be closely associated with poor renal function in patients with acute kidney injury (AKI), but whether CRP is pathogenic or a mere biomarker in AKI remains largely unclear. Impaired autophagy is known to exacerbate renal ischemia-reperfusion injury (IRI). We examined whether the pathogenic role of CRP in AKI is associated with reduction of autophagy. We mated transgenic rabbit CRP over-expressing mice (*Tg-CRP*) with two autophagy reporter mouse lines, *Tg-GFP-LC3* mice (*LC3*) and *Tg-RFP-GFP-LC3* mice (*RG-LC3*) respectively to generate *Tg-CRP-GFP-LC3* mice (*PLC3*) and *Tg-CRP-RFP-GFP-LC3* mice (*PRG-LC3*). AKI was induced by IRI. Compared with *LC3* mice, *PLC3* mice developed more severe kidney damage after IRI. Renal tubules were isolated from *LC3* mice at baseline for primary culture. OKP cells were transiently transfected with *GFP-LC3* plasmid. CRP addition exacerbated lactate dehydrogenase release from both cell types. Immunoblots showed lower LC-3 II/I ratios and higher levels of p62, markers of reduced autophagy flux, in the kidneys of *PLC3* mice compared to *LC3* mice after IRI, and in primary cultured renal tubules and OKP cells treated with CRP and H_2_O_2_ compared to H_2_O_2_ alone. Immunohistochemistry showed much fewer LC-3 punctae, and electron microscopy showed fewer autophagosomes in kidneys of *PLC3* mice compared to *LC3* mice after IRI. Similarly, CRP addition reduced GFP-LC3 punctae induced by H_2_O_2_ in primary cultured proximal tubules and in GFP-LC3 plasmid transfected OKP cells. Rapamycin, an autophagy inducer, rescued impaired autophagy and reduced renal injury *in vivo*. In summary, it was suggested that CRP be more than mere biomarker in AKI, and render the kidney more susceptible to ischemic/oxidative injury, which is associated with down-regulating autophagy flux.

## Introduction

Acute kidney injury (AKI) is characterized by rapid loss of renal function and a myriad of systemic disturbances. AKI incidence is steadily increasing over decades [1,2], and mortality [3,4] is staggeringly high in its acute phase due to limited effective definitive therapy [5,6]. Even among those who have apparent clinical recovery from AKI, there is still an estimated 25% increase in risk of progression to chronic kidney disease (CKD) and a 50% increase in mortality after more than 10 years of follow-up compared to the general population [7,8]. AKI is proposed to be an independent risk factor for the development of CKD and end-stage renal disease (ESRD) [7,9]. Pathologically, AKI is characterized by tubular injury and cell death mainly in the form of necrosis and apoptosis. Tubular epithelial cell regeneration has been reported to determine the progression of repair in AKI, which is regulated by the balance of cell proliferation and apoptosis [10,11]. Increased apoptosis was shown to inhibit tubular cell regeneration and delay recovery of renal function after AKI [12,13]. Suppression of apoptosis could promote regeneration and promote recovery from AKI [13,14].

Macroautophagy (referred to as autophagy hereafter) has been implicated with numerous pathologies. Autophagy is an evolutionarily conserved catabolic “self-eating” process that sequesters cytoplasmic components into vesicles called autophagosomes which then fuse with lysosomes, to degrade and recycle unnecessary cellular components [15–17]. Autophagy is induced in various pathological conditions and is adaptive and protective for cell survival [18,19]. Dysregulated autophagy leads to self-killing and cell death [20–22]. Defective autophagy flux was shown in various kidney diseases [23,24]. Autophagy deficiency in the proximal tubule with conditional autophagy-related gene deletion exacerbates AKI [25,26]. Enhancing autophagy may be a novel therapeutic approach to minimize kidney injury and slow CKD progression [25]. At the organelle level, sequestration of damaged lysosomes through autophagy is indispensable for balanced cellular and tissue homeostasis, lysosomal biogenesis and recovery from kidney injury [27]. Our group has recently reported that activation of autophagy is renoprotective and mitigates progression of AKI to CKD [28].

C-reactive protein (CRP), a member of pentraxin family, has high affinity to phosphocholine residues, which helps with handling of necrotic [29] and apoptotic [30] cells. It also binds to other autologous and extrinsic ligands. CRP is recognized by C1q and potently activates the classical complement pathway following aggregation or binding to macromolecular ligands [31]. Known as an acute-phase protein, it is found to become elevated rapidly in various inflammatory states. It is mostly studied in cardiovascular diseases [32–34]. Clinically, serum levels of CRP are increased in patients with AKI [11,35–39], but there have been very few studies addressing the role of CRP in kidney disease [40,11]. CRP has been shown to accelerate kidney injury in AKI animal models by impairing G1/S cell cycle or unbalancing macrophage activation and FcγR expression [11,41]. The current study explores whether and how CRP exacerbates IRI-induced AKI by down-regulating autophagy.

## Materials and methods

### Clinical data of AKI patients

Based on RIFLE criteria [42], a total of 190 non-sepsis AKI patients were included from the First Affiliated Hospital of Nanjing Medical University, Nanjing, China between November 2013 and January 2015. Patients with diabetes, cancer and CKD were excluded. The clinical protocol was approved by the Institutional Review Board of the First Affiliated Hospital of Nanjing Medical University (2016-SR-013). All the patient records/information were anonymized and de-identified prior to analysis. Data of serum CRP, serum creatinine (SCr), BUN and other parameters at the time of AKI diagnosis (referred at acute phase of AKI based on RIFLE criteria) were obtained from the hospital medical records system. Among the 190 AKI patients, 28 had sequential blood data (S1 Table). By 14 days after AKI diagnosis regardless of whether AKI patients recovered or not, we divided 28 patients into two groups: complete recovery, partial recovery or no recovery based on KDIGO criteria [43]. The clinical characteristics of these patients are shown in S1 Table.

### Animal models and experiments

A transgenic mouse [44] with over-expression of rabbit-CRP driven by promoter/regulatory region of phosphoenolpyruvate carboxykinase was kindly provided by Dr. Philip Shaul and Dr. Chieko Mineo (University of Texas Southwestern Medical Center, Texas, USA). This mouse line when fed normal chow has elevated baseline CRP levels [45–47]. A transgenic reporter mouse with *GFP-LC3* [48,49] was kindly gifted from Dr. Noboru Mizushima (Tokyo Medical and Dental University, Tokyo, Japan). An enhanced *GFP (eGFP)–LC3* is over-expressed by the CAG promoter (cytomegalovirus immediate-early (CMVie) enhancer and chicken β-actin promoter) [48,50]. The second transgenic reporter mouse is double *LC3* reporter mouse (*RFP-LC3 and GFP-LC3*) driven by CAG promoter [19] which was kindly provided by Dr. Joseph Hill (University of Texas Southwestern Medical Center, Texas, USA) [51,19]. All mouse lines were cross-mated with *WT* mice *129 S1/SVlmJ* (*129 SV*) for ~5 generations. After that, these mouse lines with a *129 SV* background were cross-mated with each other to obtain *Tg-CRP-GFP-LC3* mice (*PLC3*), *GFP-LC3* transgenic mice (*LC3*), *Tg-CRP-RFP-GFP-LC3* mice (*PRG-LC3*) and *RFP-GFP-LC3* mice (*RG-LC3*) for surgery.

For AKI surgery, ketamine was injected intraperitoneally for anesthesia. AKI induction was performed in 3 month-old mice by bilateral ischemia reperfusion injury (Bi-IRI) using established methods from our laboratory [52]. After 45 minutes of bilateral ischemia, the kidneys were reperfused and termination was conducted in 1, 2, or 7 days after IRI. For termination study, isoflurane was inhaled. Each experimental group, there were 4 mice at different time points.

To up-regulate autophagy activity, rapamycin (LC Laboratories, MA, USA) or bafilomycin A1 (Sigma-Aldrich, St. Lois, MO) was prepared as previously reported [53], and both injected at a dose of 1 mg/kg/day into *PLC3* mice intraperitoneally for three days before ischemia injury, followed by 1 day reperfusion. There were 4 mice in each group. Our animal protocol was approved by the Institutional Animal Care and Use Committee at the University of Texas Southwestern Medical Center, Texas, USA.

### Blood and kidney samples collection from mice

Blood samples were collected two days after surgery when mice were anesthetized with isoflurane for termination study, and serum was separated and stored at −80°C until analysis. Previously published methods were used for urinary and serum biochemistry measurements [54]. For histology study, kidneys were isolated at 1, 2, and 7 days after IRI and sliced. The kidney slices were fixed with 4% paraformaldehyde and embedded in paraffin blocks or Optimal Cutting Temperature (O.C.T) compound (Sakura, CA, USA) for histology or immunohistochemistry studies; the remaining parts of kidneys were snap-frozen in liquid N_2_ and stored at −80°C for future studies.

### Measurement of mouse serum CRP

To investigate the serum levels of both exogenous and endogenous CRP at baseline and one day post-IRI in *LC3* and *PLC3* mice, ELISA assays were used to measure the transgenic (rabbit) CRP levels with methods described previously [45,46], and the endogenous (mouse) CRP levels with a commercial ELISA kit (Life Diagnostics, Inc., PA, USA) according to manufacturer’s instruction.

### Measurement of mouse serum and urine creatinine

Using previously published methods [28], serum and urine creatinine concentrations were measured using a P/ACE MDQ Capillary Electrophoresis System and photodiode detector (Beckman-Coulter, Fullerton, CA).

### Mouse kidney histology, immunohistochemistry and immunoblotting

Four μm sections of paraffin embedded kidney tissues were stained with Hematoxylin and Eosin (H&E), Periodic acid–Schiff (PAS), and Trichrome. Tissue damage was examined in a blinded manner and scored as percentage of damaged tubules: 0, no damage; 1, <25%; 2, 25–50%, 3, 50–75%, 4, >75% [15]. To evaluate renal fibrosis, the fibrotic area and fibrosis intensity in Trichrome-stained kidney sections were quantified with Image J program using published methods by an investigator blinded to the conditions [54]. To further quantify fibrillary collagen accumulation in the kidney, the kidney sections were stained with Sirius Red/Fast Green Kit (Chondrex, Inc., Redmond, WA) following the kit’s instructions [28]. Apop Tag red *in situ* apoptosis detection kit (EMD Millipore, MA, USA) was used for terminal deoxynucleotidyl transferase dUTP nick end labeling (TUNEL) assay following the manufacturer’s protocols. Immunohistochemistry and immunoblotting were performed as previously described [55,52,56]. The primary antibodies used in this experiment are listed below: rabbit LC3 antibody (Novus Biologicals, CO, USA), mouse monoclonal p62/SQSTM1 antibody (Novus Biologicals, CO, USA), goat neutrophil gelatinase-associated lipocalin (NGAL) antibody (R & D, MN, USA), mouse monoclonal anti-β-actin (Sigma Aldrich, MO, USA).

### Immunoprecipitation

To test binding of Beclin 1 to Bcl-2, co-immunoprecipitation was performed using a mouse monoclonal Bcl-2 antibody (Santa Cruz Biotechnology, CA, USA) and immunoblotted using the mouse monoclonal Bcl-2 antibody and a mouse monoclonal Beclin 1 antibody (Santa Cruz Biotechnology, CA, USA), respectively, as described [57,58].

### Transmission electron microscopy

Kidney slices were prepared from mouse kidneys and fixed overnight with 2.5% glutaraldehyde and 2% paraformaldehyde in cacodylate buffer (0.1 M, pH 7.4). The ultrathin sections were cut on an ultra cryomicrotome (Ultra Microtome Reichert Ultracut E; Leica Microsystems, Wetzlar, Germany) and were visualized with Jeol 1200 EX transmission electron microscope (TEM) (Jeol Ltd., Akishima, Japan) in a blind manner as described in literatures [28].

### Primary culture of renal tubules

Under sterile conditions, renal proximal tubules were isolated from collagenase-digested cortical fragments of *LC3* mouse kidneys following previously described protocols with modification [59]. Briefly, renal cortices were dissected visually on ice and slices were transferred to Hanks’ balanced salt solution (HBSS) with 0.1% (wt/vol) type-2 collagenase and 100 μg/ml soybean trypsin inhibitor and digested for 45 min at 37°C. After digestion, the supernatant was passed through two nylon sieves (pore size 180 μm and 80 μm, Millipore, USA). The 80-μm sieve yielded a large number of long proximal tubule (PT) fragments without substantial contamination of other nephron segments or glomeruli. The PTs present in the solution were centrifuged for 5 min at 4°C and 170 *g*, washed, and then re-suspended into the appropriate amount of culture medium. The PT fragments were seeded into 12-well plates and left unstirred for 48 h at 37°C and 95% air plus 5% CO_2_, after which the culture medium was changed for the first time. The medium was then replaced every 2 days. After 7 days, cell cultures were confluent monolayers and ready for *ex vivo* experiment such as H_2_O_2_, (200 μM), CRP (10 mg/ml) [11] and/or autophagy inducer (rapamycin, 250 nM) for 24 hours. Pure CRP was purchased from Millipore (CALBIOCHEM, Japan) and 30% hydrogen peroxide (H_2_O_2_) from Sigma-Aldrich (St. Louis, MO, USA). Lactate dehydrogenase (LDH) Cytotoxicity Detection Kit was purchased from TaKaRa (Takara Bio USA, Inc., Mountain View, CA, USA). The 8-hydroxydeoxyguanosine (8-OHdG) formation is a ubiquitous marker of oxidative DNA injury. The 8-OHdG concentration in culture media was determined by ELISA (OxiSelect Oxidative DNA damage, Cell BioLabs, San Diego, CA, USA) following the manufacture’s instruction to assess oxidative stress. We also seeded PTs on coverslips under the same culture conditions for immunostaining studies. The majority of renal tubules (85–90%) was identified as proximal tubules by immunostaining (S1 Fig). We conducted the *ex vivo* studies in at least three independent experiments.

### Cell culture

One type of opossum kidney cell line (OKP, a proximal tubule cell line with PTH receptor) was a kind gift from Dr. Judith Cole (University of Memphis). LDH and 8-OHdG concentration in culture media was determined by ELISA kits following the manufacture’s instruction to assess oxidative stress. The GFP-LC3 fusion plasmid was kindly provided by Dr. Mizushima N. OKP cells were maintained in high glucose DMEM medium as described [56,60]. Both primary cultured renal tubular cells and OKP cells were treated with H_2_O_2_ and/or CRP in the presence or absence of rapamycin for 24 hours. Cell lysates were prepared [61,56] and subjected to immunoblotting. Cell culture media were harvested for measurement of LDH release as a cell injury marker following the protocol we previously described [28]. The GFP-LC3 fusion plasmid was transiently transfected into OKP cells using Lipofectamine 2000 (Invitrogen, CA, USA). One day after transfection, cells were treated with H_2_O_2_, CRP and/or autophagy modulators. One day after treatment, cells were fixed, stained with Syto61 (1:200, Life technologies, OR, USA) and rhodamine-phalloidin (1:100, Cytoskeleton, CO, USA), and underwent confocal fluorescent microscopy. Then images of immunoblotting and immunostaining were used for semi-quantitative analysis according to established protocols [28]. We conducted the *in vitro* studies in at least three independent experiments.

### Statistical analyses

Data are expressed as means ± SD from at least 4 independent experiments. Statistical analysis was performed using unpaired t-test or one-way analysis of variance (ANOVA) followed by *post hoc* Newman-Keuls test when applicable. In addition, linear regression was used for correlation studies between the serum CRP levels and other parameters. All statistical analyses were performed with Prism software (Prism 5.01, GraphPad software). When the P value was ≤ 0.05, the difference was considered statistically significant.

## Results

### Serum levels of CRP are correlated with the severity of renal impairment in patients with AKI

**Fig 1:**
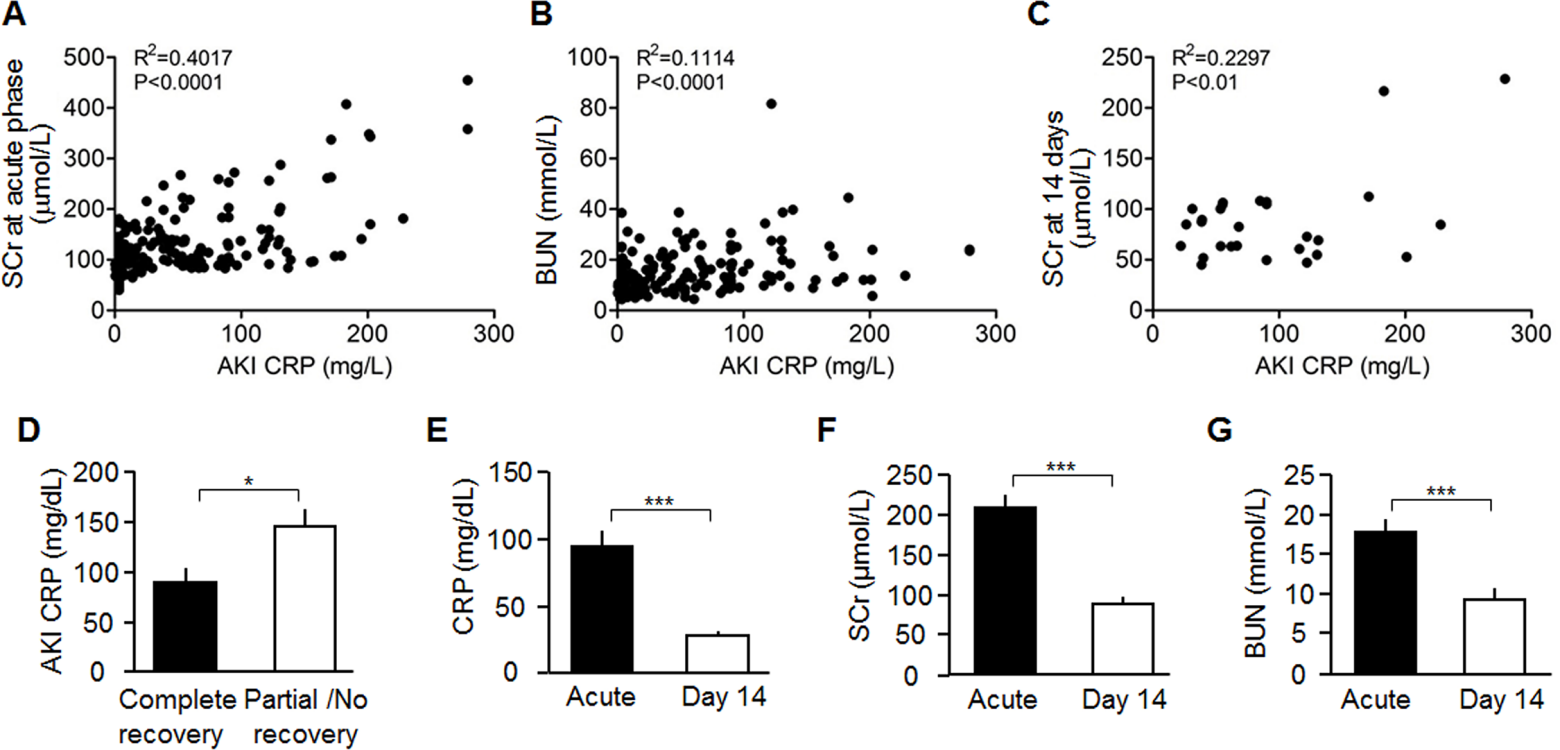
Correlation between serum CRP levels and other parameters. (A-B) Correlation between serum CRP levels and SCr/BUN levels in 190 AKI patients at acute phase of AKI. (C) Correlation between serum CRP levels at acute phase and SCr levels at 14 days after AKI diagnosis in 28 AKI patients. (D) Comparison of Serum CRP levels at acute phase between complete recovery patients and partial or no recovery patients. (E-G) Serum Levels of CRP, Cr, and BUN in AKI patients at acute phase and 14 days. Results are means ± SD from 28 AKI patients, and statistical significance was assessed by unpaired Student t-test. *: P<0.05. ***: P<0.001 between groups.

Based on the blood data from 190 AKI patients, serum CRP levels at acute phase were correlated positively with SCr and BUN (Fig 1A and 1B). Due to availability of patients’ laboratory data, only 28 AKI patients were enrolled to further analyze the correlation of serum CRP at acute phase with renal outcome at 14 days after AKI diagnosis. Serum CRP levels at acute phase were also positively correlated with SCr at 14 days (Fig 1C). We then divided 28 patients into 2 groups: complete recovery (n = 18), and without recovery including partial recovery (n = 7) and no recovery (n = 3) following published definition criteria [43]. Serum CRP levels at acute phase were found to be statistically different between these two groups (Fig 1D), indicating that relatively low serum CRP at acute phase of AKI predicts better renal recovery. At 14 days after AKI diagnosis, serum CRP levels was significantly reduced along with a decline in SCr and BUN during renal recovery (Fig 1E–1G), further supporting that serum CRP levels are associated with the severity of kidney injury.

### Serum levels of rabbit CRP and mouse CRP before and after AKI

Consistent with published data [44], serum levels of rabbit CRP were undetectable (<1 μg/mL) in *LC3* mice and were 9–21 μg/mL in *PLC3* mice at baseline. After IRI, both *PLC3* and *LC3* mice had significantly higher levels of endogenous (mouse) CRP detected by ELISA assay, compared to their own Sham group (S2A Fig). Furthermore, higher serum levels of mouse CRP were found in *PLC3* mice than *LC3* mice after IRI (S2A Fig), and the more elevation of CRP was also detected in kidney lysates of *PLC3* mice than in *LC3* post-AKI (S2B Fig), suggesting that *PLC3* mice might have more kidney injury post IRI compared to *LC3* mice. However, serum levels of transgenic (rabbit) CRP were not increased in *PLC3* mice after IRI compared with Sham group (data not shown).

### Mice with high CRP develop more severe acute kidney injury induced by IRI

**Fig 2:**
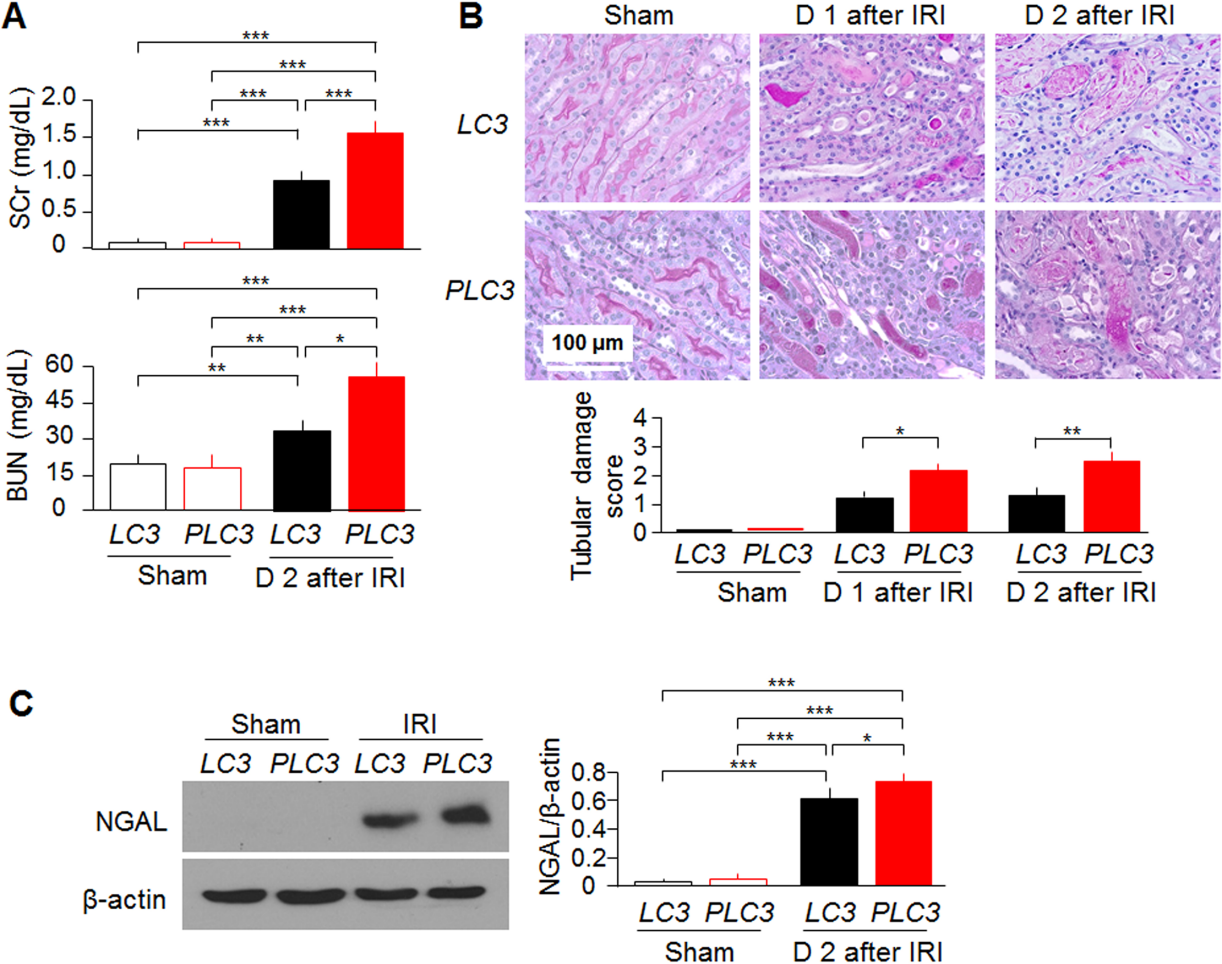
CRP exacerbates acute kidney injury *in vivo*. (A) SCr and BUN of *PLC3* mice and *LC3* mice prior to and post-IRI. (B) Representative PAS stain of kidney sections at Day 1 and Day 2 after IRI. Tissue damage was scored by the percentage of damaged tubule. (C) NGAL protein levels in the kidney lysates of *PLC3* mice and *LC3* mice prior and post-IRI. Data are expressed as means ± SD of at least 4 mice from each group and statistical significance was assessed by one-way ANOVA followed by Newman-Keuls test. *: P<0.05, **: P<0.01, ***: P<0.0001 between two groups.

**Fig 3:**
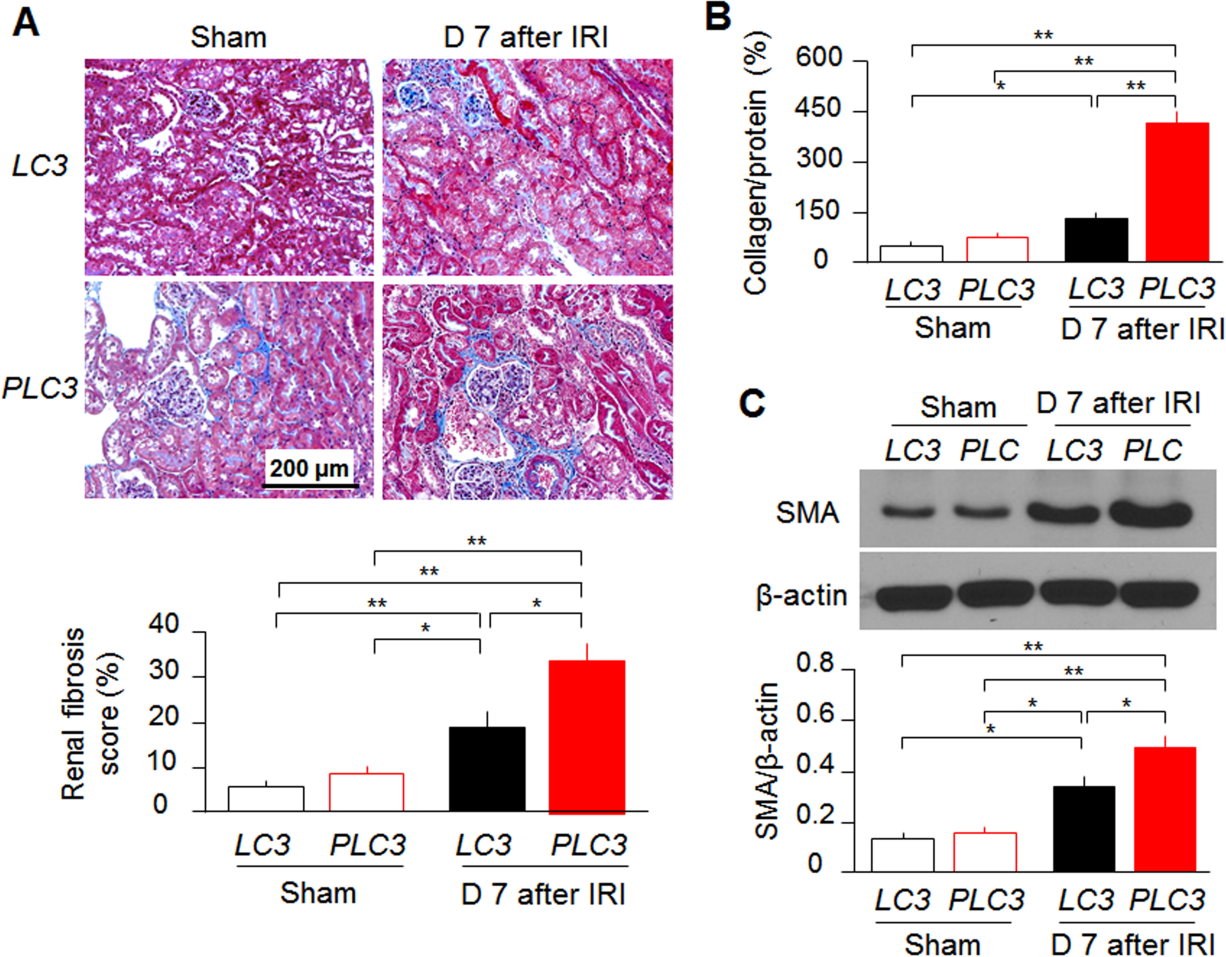
CRP induces more fibrosis Day 7 post-IRI. (A) Representative Trichrome stain of kidney sections at Day 7 after IRI. Interstitial fibrosis was scored following published protocol. (C) SMA protein levels in the kidney lysates of *PLC3* mice and *LC3* mice prior and post-IRI by western blotting. Data are expressed as means ± SD of at least 4 mice from each group and statistical significance was assessed by one-way ANOVA followed by Newman-Keuls test. *: P<0.05, **: P<0.01 between two groups.

One day after IRI induction, *PLC3* mice developed more severe AKI as evidenced by higher SCr and BUN levels than *LC3* mice (Fig 2A). The expression of NGAL, a kidney injury marker [62], was higher in kidney lysates of *PLC3* than *LC3* mice (Fig 2C). *PLC3* mice developed more severe tubular damage, identified by more tubular necrosis and casts in histologic sections at Day 1 and Day 2 post-IRI compared with *LC3* mice (Fig 2B, and S3A and S3B Fig). More tubulointerstitial fibrosis was found in Trichrome-stained kidney sections (Fig 3A), quantitative analysis of Sirius red stain (Fig 3B), and SMA expression was higher (Fig 3C) at Day 7 post-IRI in *PLC3* mice than *LC3* mice, suggesting that CRP mice are more susceptible to develop renal fibrosis after ischemic injury. Interestingly note that *PLC3* mice had a little renal fibrosis and very mild elevation of systemic blood pressure compared to *LC3* mice at baseline (S4 Fig). Currently we still do not know whether slight increase in interstitial fibrosis was due to endothelial injury or mild increase in systemic blood pressure in *PLC3* mice. We anticipate that old *PLC3* mice may have spontaneous hypertension and interstitial fibrosis.

### CRP enhances oxidative cell injury *ex vivo* and *in vitro*

**Fig 4:**
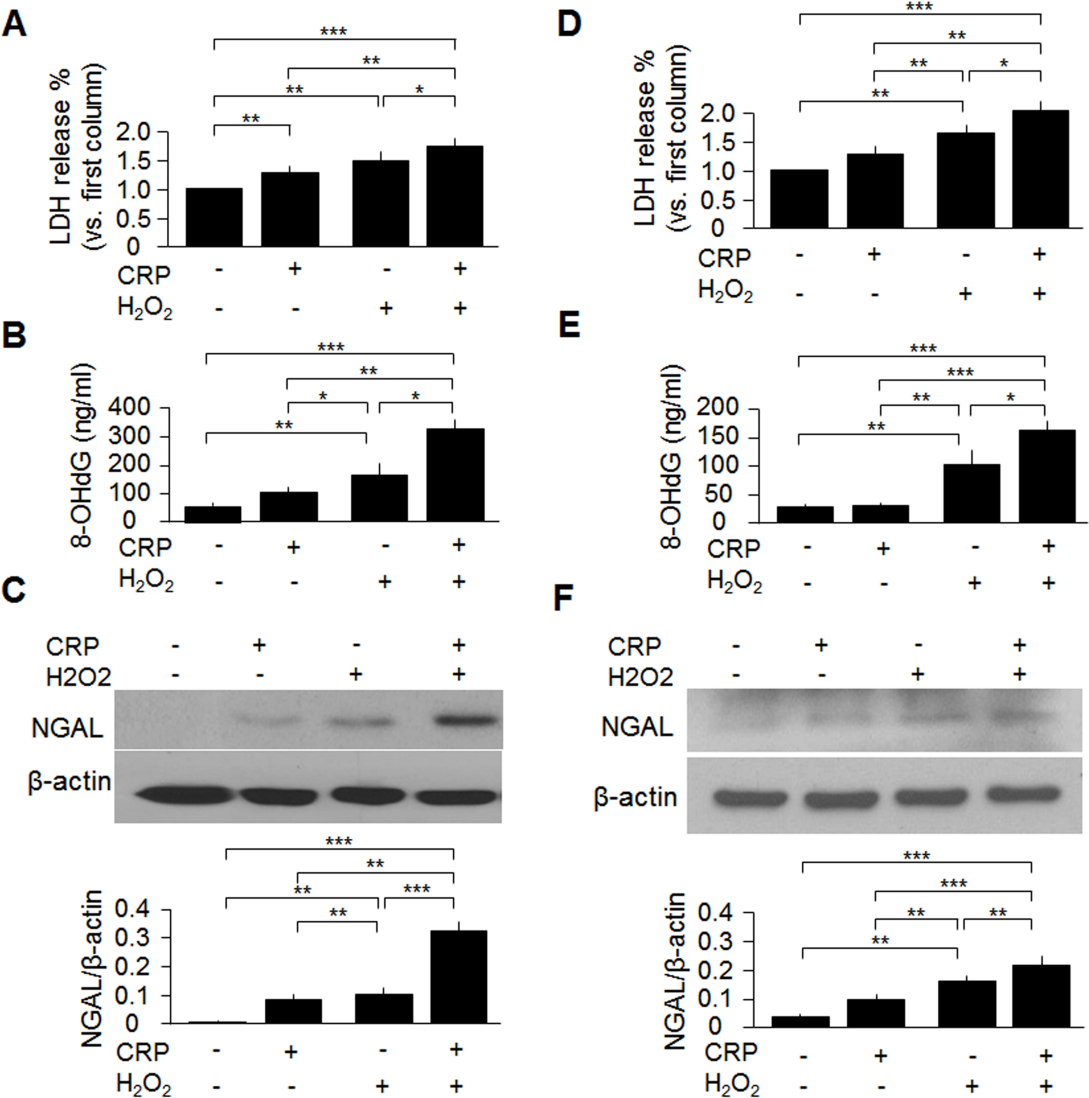
CRP treatment exacerbates oxidative stress *ex vivo* and *in vitro*. Primary cultured cells (A-C) and OKP cells (D-F) were treated with or without CRP in the presence of H_2_O_2_ (200 μM for 24 hours) or vehicle (PBS). (A, D) LDH release in primary cultured tubular cells or OKP cells with or without CRP treatment at baseline and oxidative stress. (B, E) 8-OHdG release from primary cultured tubular cells or OKP cells with or without CRP treatment at baseline and oxidative stress. (C, F) NGAL protein expression in the primary cultured tubular cells or OKP cells with or without CRP treatment at prior and post oxidative stress. Data are expressed as means ± SD of at least 3 independent experiments for each group and statistical significance was assessed by one-way ANOVA followed by Newman-Keuls test. *: P<0.05, **: P<0.01, ***: P<0.0001 between two groups.

To directly examine whether CRP exacerbates oxidative injury, we isolated renal tubules from *LC3* mice. Enriched proximal tubular epithelial cells were cultured, and treated directly with H_2_O_2_ and/or CRP. H_2_O_2_ increased LDH (a cell injury marker) and 8-OHdG (an oxidative marker) release from primary cultured renal proximal tubular epithelial cells, which was exacerbated by CRP treatment compared with vehicle treatment (Fig 4A and 4B). CRP treatment also increased NGAL expression in the cell lysates after H_2_O_2_ incubation compared to vehicle treatment (Fig 4C). Furthermore, CRP treatment elevated LDH and 8- OHdG release from OKP cells after H_2_O_2_ incubation (Fig 4D and 4E) and induced a robust increase in NGAL (Fig 4F) compared with vehicle treatment. Those *ex vivo* and *in vitro* experiments provided further evidences to support our notion that CRP exacerbates oxidative injury.

### CRP impairs autophagy flux

**Fig 5:**
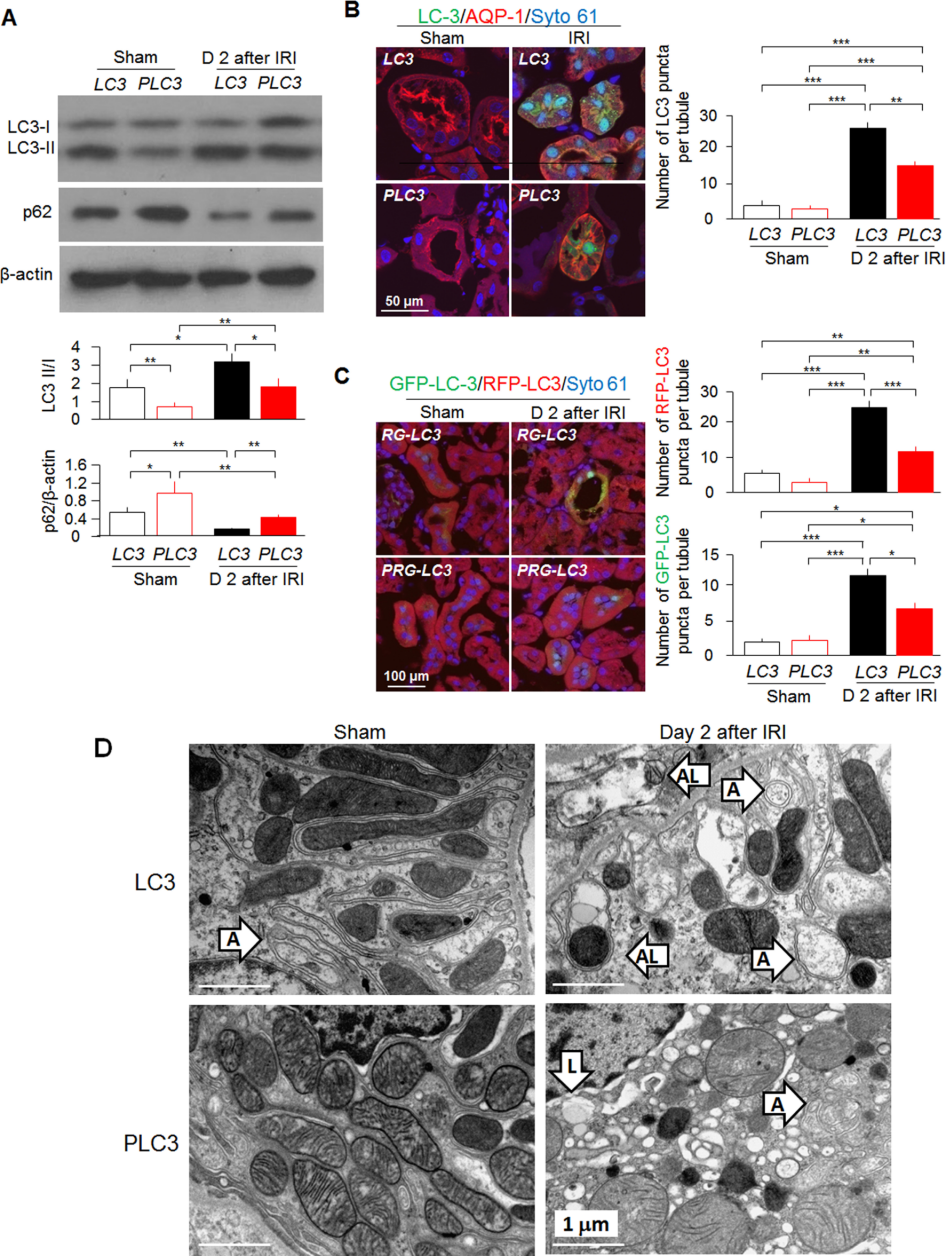
CRP impairs autophagy *in vivo*. (A) LC3 II/I and p62 levels in *PLC3* mice and *LC3* mice prior and post-IRI by immunoblotting. (B) GFP-LC3 punctae in *PLC3* mice and *LC3* mice prior and post-IRI by immunohistochemistry. (C) RFP-LC3 and GFP-LC3 punctae in *RG-LC3* mice and *PRG-LC3* mice prior and post-IRI by immunohistochemistry. (D) Representative TEM for autophagosomes and autolysosomes in the kidneys. Data are expressed as means ± SD of at least 4 mice from each group and statistical significance was assessed by one-way ANOVA followed by Newman-Keuls test. *: P<0.05, **: P<0.01, ***: P<0.0001 between two groups. A: autophagy; AL: autolysosome; and L: Lysosome.

**Fig 6:**
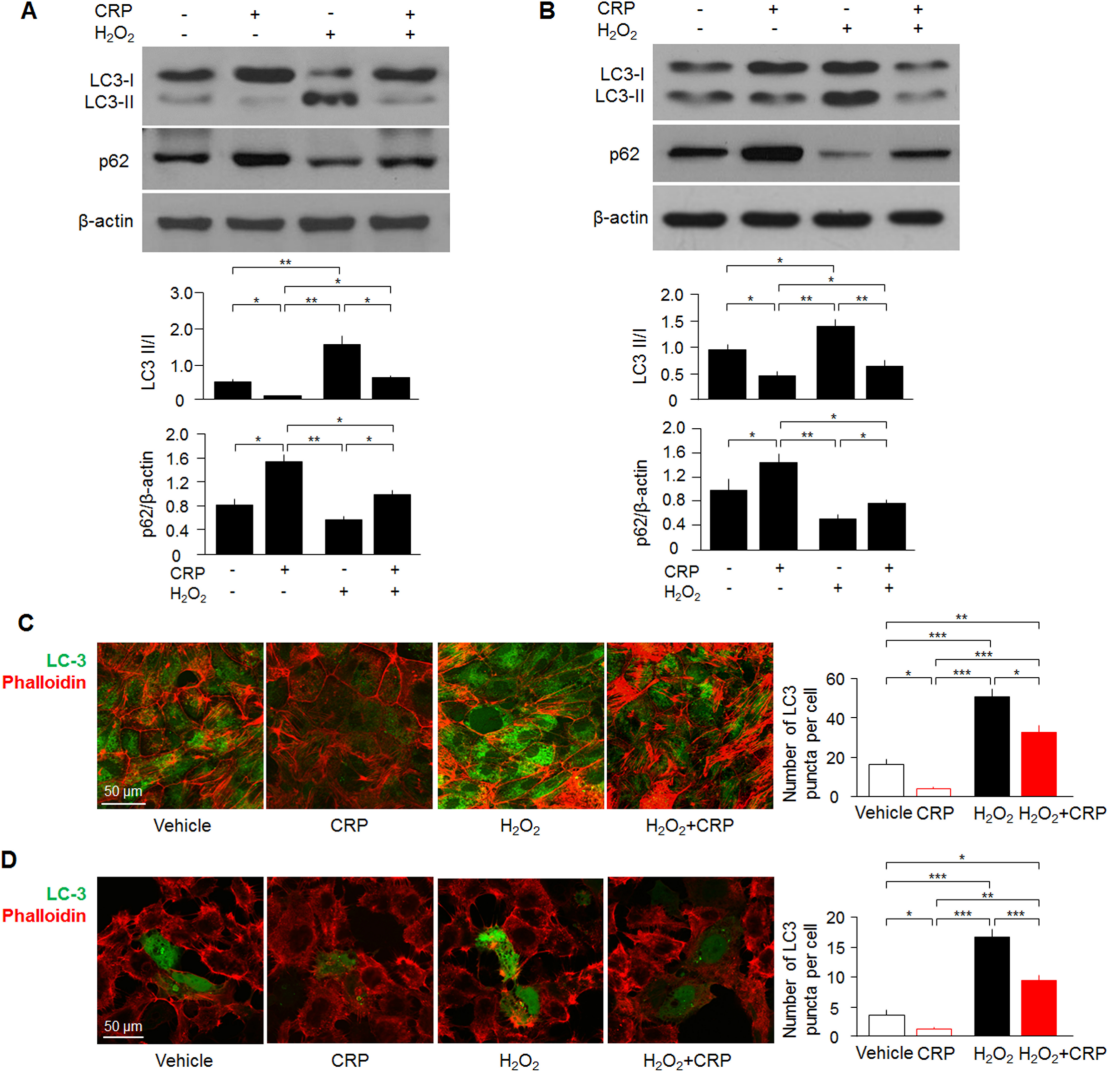
CRP suppresses autophagy *ex vivo* and *in vitro*. (A) LC3 II/I and p62 levels in primary cultured tubular cells with or without CRP treatment at baseline and oxidative stress by immunoblotting. (B) LC3 II/I and p62 levels in OKP cells with or without CRP treatment by immunoblotting. (C) GFP-LC3 punctae in primary cultured tubular cells by immunohistochemistry. (D) GFP-LC3 punctae in OKP cells by immunohistochemistry. Data are expressed as means ± SD of at least 3 independent experiments for each group and statistical significance was assessed by one-way ANOVA followed by Newman-Keuls test. *: P<0.05, **: P<0.01, ***: P<0.0001 between two groups.

Since autophagy dysfunction worsens kidney injury in various AKI models [26,28], we determined two classical autophagy markers—microtubule-associated protein 1A/1B-light chain 3 (LC3) and p62 [63] to examine autophagy flux in the kidney, cultured proximal tubules and OKP cells. LC3-I, a cytosolic form of LC3, is conjugated to phosphatidylethanolamine to form LC3-phosphatidylethanolamine conjugate (LC3-II), which is recruited to autophagosomal membranes during autophagy process. Autophagosomes fuse with lysosomes to form autolysosomes, and intra-autophagosomal components including LC3-II are degraded by lysosomal hydrolases [26,28,63]. Thus, detecting LC3II/I ratio by immunoblotting or examining autophagosomes by immunofluorescence have become a reliable method for monitoring autophagy-related processes. We found lower ratios of LC3 II/I and higher levels of p62 in the kidney lysates at baseline and after AKI in *PLC3* mice compared with *LC3* mice (Fig 5A). Immunostaining showed that *PLC3* mice had less induced GFP-LC3 punctae than *LC3* mice (Fig 5B), indicating fewer autophagosomes in mice with high CRP. To examine if decreased GFP-LC3 punctae are due to more GFP-LC3 trapping in autolysosomes (GFP fluorescence is quenched in acidic environments), we used double LC3 reporter mice which red RFP-LC3 signal that is not bleached in autolysosomes. We found that both RFP-LC3 punctae and GFP-LC3 punctae were less in *PRG-LC3* mice compared with *RG-LC3* mice after AKI (Fig 5C), indicating that CRP blunted autophagy activation by IRI which was confirmed by electron microscopic images (Fig 5D). Furthermore, decreased ratios of LC3 II/I and increased levels of p62 were also found in cell lysates of both cultured proximal tubular cells *ex vivo* and OKP cells *in vitro* (Fig 6A and 6B). Fewer punctae of LC3 were found in primary cultured proximal tubular cells from *LC3* mice and OKP cells transiently transfected with GFP-LC3 after H_2_O_2_ treatment compared with vehicle treatment (Fig 6C and 6D), indicating that CRP suppresses H_2_O_2_-induced autophagy flux.

### CRP induces Beclin 1 binding to anti-apoptotic Bcl-2

**Fig 7:**
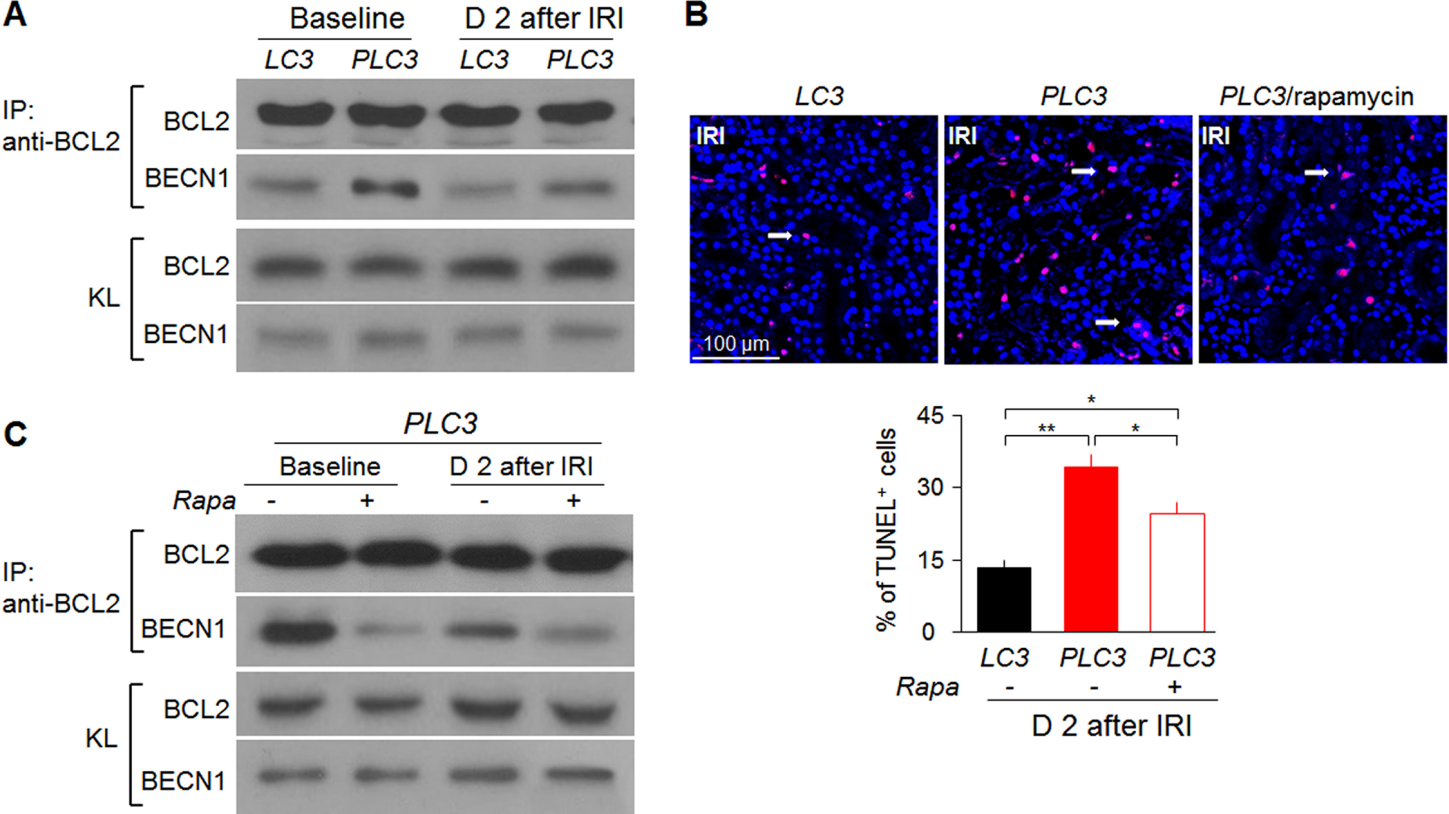
CRP induces apoptosis and suppresses autophagy. (A) Binding of Beclin 1 to Bcl-2 by co-IP in *LC3* mice and *PLC3* mice post-IRI. (B) Number of TUNEL positive cells in *LC3* mice, *PLC3* mice and *PLC3* mice with rapamycin injection post-IRI. Data are expressed as means ± SD of 4 mice from each group and statistical significance was assessed by one-way ANOVA followed by Newman-Keuls test. *: P<0.05, **: P<0.01 between two groups. (C) Binding of Beclin 1 and Bcl-2 by co-IP in *PLC3* mice post-IRI with or without rapamycin injection before surgery. KL: whole kidney lysates.

Dissociation of Bcl-2 and Beclin 1 is an important mechanism for activating autophagy under nutrient deprivation [57,64]. In contrast, nutrient excess increases Bcl-2 binding to Beclin 1 and inhibits autophagy. To define the molecular mechanism by which CRP down-regulates autophagy, we performed co-IP to semi-quantitatively measure Bcl-2 and Beclin 1 complexes. *PLC3* mice had more Beclin 1 bound to Bcl-2 (Fig 7A) than *LC3* mice at baseline, which indicated that CRP might suppress autophagy by inhibiting Beclin 1 release from Bcl-2/Beclin 1 complexes. Consistent with decreased autophagy and increased Bcl-2/Beclin 1 binding, there was more apoptosis as shown by more TUNEL positive cells (Fig 7B) in the kidney of *PLC3* mice compared to *LC3* mice. Rapamycin also helped Beclin 1 escape from Bcl-2/Beclin 1 complexes to induce autophagy (Fig 7C), and ameliorated apoptotic cell death documented by lower TUNEL positive cells in *PLC3* mice after AKI (Fig 7B).

### Rapamycin rescues CRP-reduced autophagy and ameliorates AKI in *PLC3* mice

**Fig 8:**
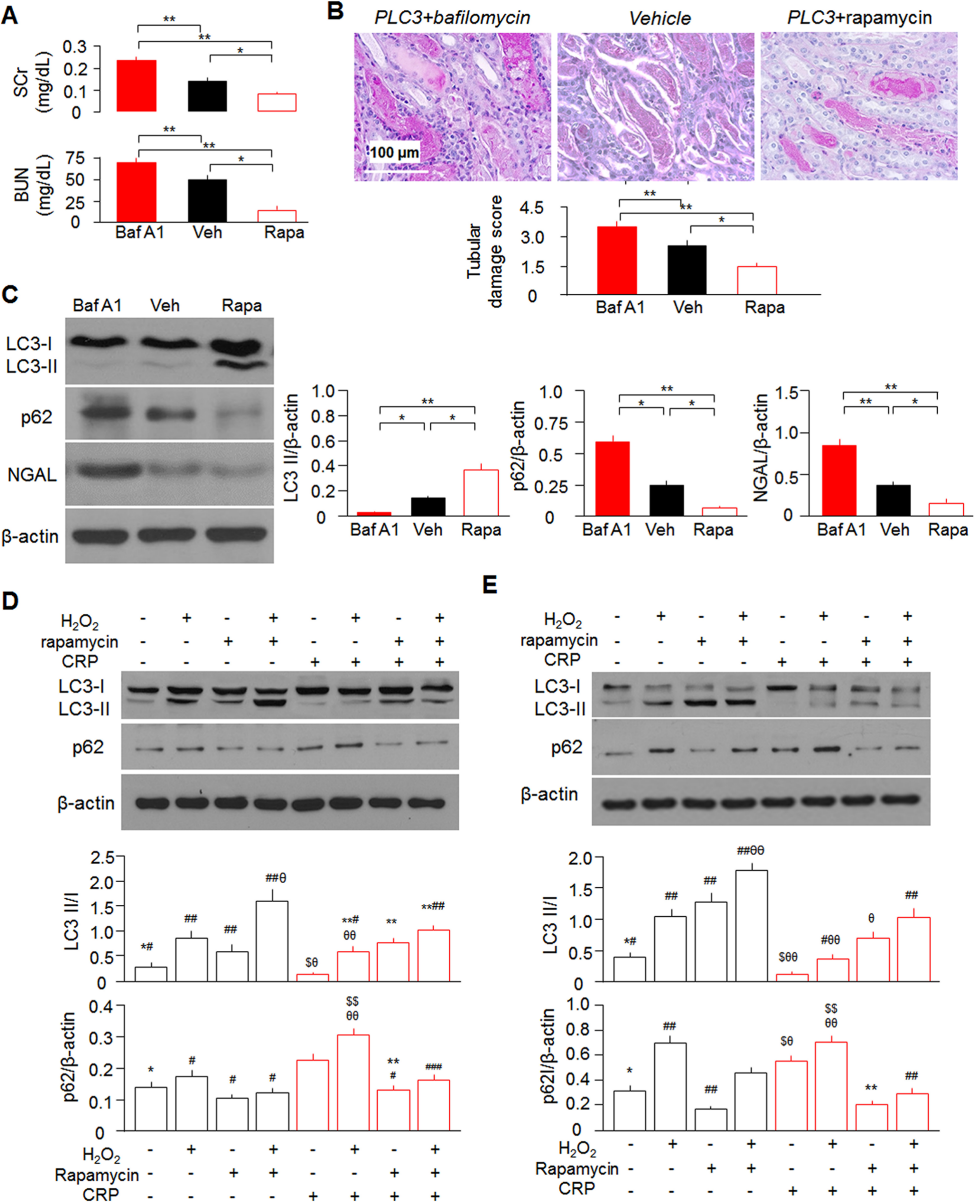
Autophagy modulators alter autophagic flux and severity of kidney injury in IRI model. (A) Rapamycin reduces SCr in *PLC3* mice post-IRI. (B) Representative PAS stain of kidney sections of *PLC3* mice with Baf A1 or rapamycin (rapa) or vehicle (Veh) injection at first day post IRI. Tubular damage was scored by the percentage of damaged tubule. (C) Rapamycin injection rescues autophagy flux and reduces NGAL expression in *PLC3* mice post-IRI. Data expressed as means ± SD of 4 mice from each group and statistical significance was assessed by one-way ANOVA followed by Newman-Keuls test. *: P<0.05, **: P<0.01 between two groups for A ~ C. Rapamycin treatment for 24 hours rescues autophagy flux and CRP/H_2_O_2_-induced cytotoxicity *ex vivo* (D) or *in vitro* (E). Data are expressed as means ± SD of at least 3 independent experiments for each group and statistical significance was assessed by one-way ANOVA followed by Newman-Keuls test for D and E. *: P<0.05, **: P<0.01 vs. CRP group; ^#^: P<0.05, ^##^: P<0.01, ^###^: P<0.0001 vs. CRP + H_2_O_2_ group; ^$^: P<0.05, ^$ $^: P<0.05 vs. CRP + rapamycin group; ^θ^: P<0.05, ^θθ^: P<0.01 vs. CRP + rapamycin + H_2_O_2_ group.

To gain direct evidence to support the *in vivo* effect of autophagy on CRP-associated severe kidney injury in IRI model, we pre-treated mice with rapamycin to upregulate or with bafilomycin A1 (Baf A1) to downregulate autophagy flux. Interestingly and importantly, rapamycin improved tubular damage, better preserved renal function and decreased NGAL expression in the kidney of AKI mice; whereas Baf A1 had opposite effect (Fig 8A–8C, and S5 Fig), further indicating that CRP-worsened kidney dysfunction and histological alteration in IRI model is associated with down-regulation of autophagy in the kidney. Rapamycin attenuated CRP-induced down-regulation of autophagy as evidenced by an increased LC3 II/I ratio and reduced p62 levels in the kidney, cultured proximal tubules and OKP cells (Fig 8C–8E). Furthermore, rescued autophagy activity by rapamycin was able to overwrite CRP-promoted cell injury induced by H_2_O_2_ (Fig 8D and 8E).

## Discussion

Consistent with previous clinical data [11], we found that AKI patients had increased levels of serum CRP during acute phase regardless of etiology. Elevated serum CRP levels were positively correlated with the levels of SCr and BUN at acute phase and with SCr at 14 days after AKI diagnosis. These clinical observations indicate a close link between CRP and AKI. A larger and long-term longitudinal study is required to confirm our findings. Emerging evidence showed that CRP is not only a biomarker, but also a contributor to AKI, because CRP was reported to promote AKI by enhancing inflammation, shifting the balance of macrophage activation and FcγR expression towards a detrimental portfolio[41], or impairing G1/S-dependent tubular epithelial cell regeneration [11]. Therefore, CRP is not only a biomarker, but also a pathogenic intermediate for AKI. Our results confirm the pathogenic model. We found that *PLC3* mice developed more severe AKI compared with *LC3* mice, which is consistent with published findings in various animal models including obstructive nephropathy [65], diabetic kidney disease [40] and ischemia-induced kidney injury [41]. We showed *PLC3* mice had more apoptotic cells than *LC3* mice post IRI, but whether other types of cell death including programmed necrosis can be induced by combinational effect of CRP and H_2_O_2_ is elusive. In addition, we will further illustrate whether H_2_O_2_ induces endoplasmic reticulum stress and consequently modulates autophagy and whether CRP blunts this upregulation and exacerbates H_2_O_2_-induced cytotoxicity.

Autophagy is an evolutionarily conserved catabolic process for terminal degradation or recycling of cytoplasmic components and serves a defense mechanism to protect and maintain normal function of cells [66–68]. Defective autophagy has been reported to render the kidney vulnerable to ischemic injury and nephrotoxicity [23,69,15,70]. Restoration of autophagy was shown to be renoprotective [28]. In the present study, we first found that *PLC3* mice developed more severe IRI-induced AKI with down-regulated autophagic flux compared with *LC3* mice. *Ex vivo* and *in vitro* data further confirmed that CRP impaired autophagy. Apoptosis plays a role in the pathogenesis of AKI [12]. The autophagy/apoptosis toggle switch is regulated by Bcl-2/Beclin-1 complex [71,64]. Bcl-2 not only functions as an anti-apoptotic protein, but also as an anti-autophagic protein via its inhibitory interaction with Beclin 1. In the absence of Bcl-2 binding, Beclin 1 induces excessive autophagy. But, when Bcl-2 binds to Beclin 1, autophagy activity is inhibited. So the Bcl-2/Beclin1 complex can be regarded as a brake on controlling autophagy activity [64]. Here, we found that autophagy was suppressed and switched to apoptosis in *PLC3* mice after AKI, possibly because there was more Bcl-2/Beclin1 complex formation. Taken together, our data indicate that CRP exacerbates AKI by down-regulating autophagy and activating apoptosis.

To explore whether autophagy deficiency induced by CRP is involved in AKI development, we applied rapamycin, a well-known autophagy inducer *in vivo*, *ex vivo* and *in vitro* to test whether it could ameliorate kidney injury. Interestingly, pretreatment of rapamycin for three days significantly reduced SCr and BUN, and attenuated histologic renal tubular damage one day after IRI. Rapamycin treatment, autophagy inducer could restore impaired autophagy induced by CRP *in vivo*, *ex vivo* and in vitro. But bafilomycin A1, autophagy suppressor exerts opposite action and worsened IRI-induced kidney injury in CRP overexpression mice. These indicate that autophagy inducer can attenuate kidney damage in the presence of high CRP, heralding it as a promising therapy for AKI patients with significantly elevated serum levels of CRP.

There was more interstitial fibrosis and higher expression of SMA in the kidneys of *PLC3* mice 7 days after IRI compared with *LC3* mice. The mechanisms of higher renal fibrosis in *PLC3* mice may be multifactorial. One is that *PLC3* mice had more severe kidney damage during the acute phase compared with *LC3* mice, which should lead to more renal fibrosis. Secondly, as demonstrated by us and others [28,72], defective autophagy is associated with abnormal fibrosis; CRP-induced down-regulated autophagy might be another potential mechanism of enhanced fibrosis observed in *PLC3* mice. More work with administration of CRP post AKI is needed to confirm if CRP promotes renal fibrosis.

In conclusion, the pathogenic role of CRP in cardiovascular diseases has been well established, but its effects on renal injury are relatively understudied. Our human study showed that elevated serum CRP levels were correlated positively with renal function both at acute phase of AKI and 14 days of acute phase. Moreover, our animal study showed that CRP gene overexpression in mice suppresses autophagy and renders the kidney more susceptible to ischemic injury. The enhancement of kidney injury by CRP is associated with down-regulation of autophagic flux, which may be a therapeutic target for AKI patients. The long-term effects of CRP on tubulointerstitial fibrosis and AKI progression to CKD merits further investigation.

## Supporting information

S1 Table Clinical characteristics of AKI patients. (DOCX)

S1 Fig Identification of cell type of the primary cultured renal tubular epithelial cells. Primary cultured renal tubular epithelial cells on coverslips were stained with rabbit AQP-1 antibody (Millipore, MA, USA) to identify renal proximal tubules (red) (A), rabbit NCC antibody (kind gift from Dr. Alicia A. Mc Donough) to identify renal distal tubules (red) (B), goat THP antibody (Santa Cruz, CA, USA) to identify Henle’s loops (red) (C) and rabbit calbindin D28k antibody (Swant, Switzerland) predominantly to identify distal renal tubules (red) (D) respectively. Phalloidin was stained blue and LC3-GFP puncta was shown as green (A-D). Overall, more than 85% of the cells were AQP-1 positive, which is a marker of proximal renal tubules. Scale bar = 100 μm.
(TIF)

S2 Fig Serum and kidney CRP levels detected by ELISA and western blotting respectively. (A) Mouse serum CRP levels were found increased by ELISA in both *LC3* and *PLC3* mice after IRI-AKI compared to Sham group respectively. *PLC3* mice had even higher mouse CRP levels after IRI compared with *LC3* mice. *: P<0.05, **: P<0.01. (B) Western blotting analysis detected similar results in kidney tissues with serum data by ELISA. It also showed that *PLC3* mice had higher CRP expression in the kidney lysates at baseline compared with *LC3* mice, which might be due to primary antibody’s non-specific binding to both mouse and rabbit CRP. Data are expressed as means ± SD of at least 4 mice from each group and statistical significance was assessed by one-way ANOVA followed by Newman-Keuls test. *: P<0.05, **: P<0.01, ***: P<0.0001 between two groups.
(TIF)

S3 Fig Kidney histology in mice of IRI-induced AKI. (A) Representative H & E stain of the kidney sections. Scale bar = 500 μm. (B) Representative PAS stain of kidney sections. Scale bar = 250 μm.
(TIF)

S4 Fig Systolic blood pressure in *LC3* and *PLC3* mice. Blood pressure was measured by tail-cuff method in wake condition with MC4000 Multichannel System (Hatteras Instruments, Cary, North Carolina). Data are expressed as means ± SD of at least 4 mice from each group and statistical significance was assessed by unpaired Student t-test. *: P<0.05 between two groups.
(TIF)

S5 Fig Kidney histology in mice of IRI-induced AKI. Representative H & E (upper panel, scale bar = 100 μm) stains and PAS (bottom panel, scale bar = 250 μm) stains on kidney sections of *PLC3* mice pre-treated with bafilomycin A1, vehicle or rapamycin for 3 days followed by IRI for 24 hours.
(TIF)
