# Supplementary material for: Downregulation of autophagy is associated with severe ischemia-reperfusion-induced acute kidney injury in overexpressing C-reactive protein mice
Source: PLoS One. 2017 Sep 8;12(9):e0181848. doi: 10.1371/journal.pone.0181848 (PMC5590740; doi:10.1371/journal.pone.0181848)
Supplement: S1 Table — (DOCX) [file pone.0181848.s001.docx]

**Parameter** ATN Rhabdomyolysis Nephrotoxin

181

4

5

4

67

Number

Gender (n)

Female

Male

Age (years)

1

3

1

114

36±8

35±3

65±1
