# Supplementary figures and images for: Downregulation of autophagy is associated with severe ischemia-reperfusion-induced acute kidney injury in overexpressing C-reactive protein mice
Source: PLoS One. 2017 Sep 8;12(9):e0181848. doi: 10.1371/journal.pone.0181848 (PMC5590740; doi:10.1371/journal.pone.0181848)

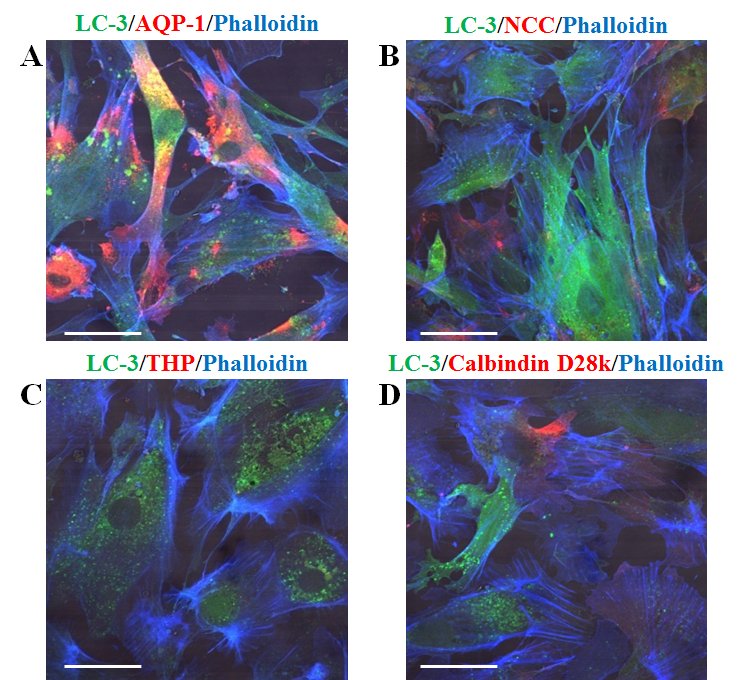

Supplement: S1 Fig — Primary cultured renal tubular epithelial cells on coverslips were stained with rabbit AQP-1 antibody (Millipore, MA, USA) to identify renal proximal tubules (red) (A), rabbit NCC antibody (kind gift from Dr. Alicia A. Mc Donough) to identify renal distal tubules (red) (B), goat THP antibody (Santa Cruz, CA, USA) to identify Henle’s loops (red) (C) and rabbit calbindin D28k antibody (Swant, Switzerland) predominantly to identify distal renal tubules (red) (D) respectively. Phalloidin was stained blue and LC3-GFP puncta was shown as green (A-D). Overall, more than 85% of the cells were AQP-1 positive, which is a marker of proximal renal tubules. Scale bar = 100 μm. (TIF) [file pone.0181848.s002.tif]

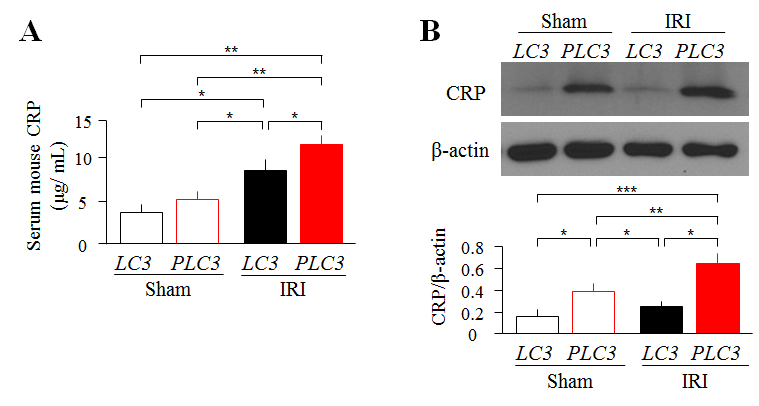

Supplement: S2 Fig — (A) Mouse serum CRP levels were found increased by ELISA in both LC3 and PLC3 mice after IRI-AKI compared to Sham group respectively. PLC3 mice had even higher mouse CRP levels after IRI compared with LC3 mice. *: P<0.05, **: P<0.01. (B) Western blotting analysis detected similar results in kidney tissues with serum data by ELISA. It also showed that PLC3 mice had higher CRP expression in the kidney lysates at baseline compared with LC3 mice, which might be due to primary antibody’s non-specific binding to both mouse and rabbit CRP. Data are expressed as means ± SD of at least 4 mice from each group and statistical significance was assessed by one-way ANOVA followed by Newman-Keuls test. *: P<0.05, **: P<0.01, ***: P<0.0001 between two groups. (TIF) [file pone.0181848.s003.tif]

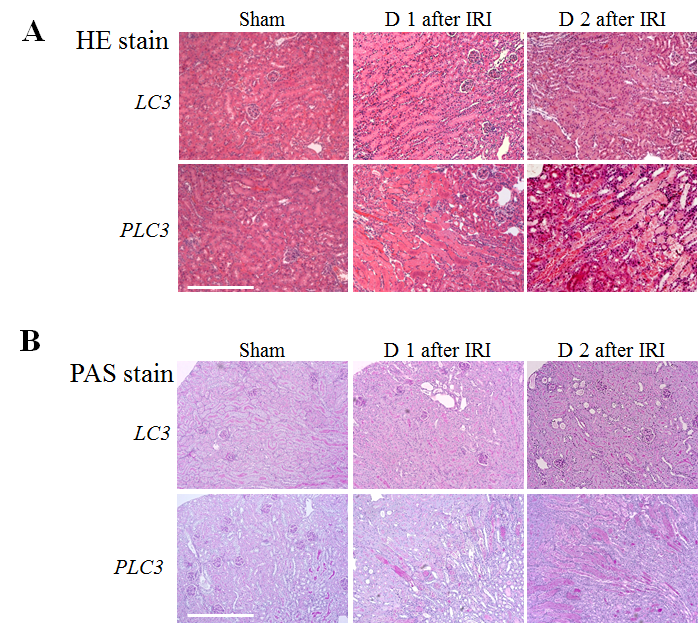

Supplement: S3 Fig — (A) Representative H & E stain of the kidney sections. Scale bar = 500 μm. (B) Representative PAS stain of kidney sections. Scale bar = 250 μm. (TIF) [file pone.0181848.s004.tif]

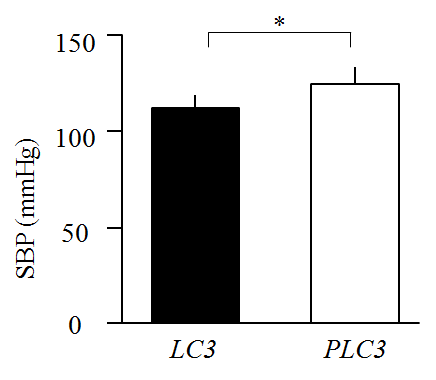

Supplement: S4 Fig — Blood pressure was measured by tail-cuff method in wake condition with MC4000 Multichannel System (Hatteras Instruments, Cary, North Carolina). Data are expressed as means ± SD of at least 4 mice from each group and statistical significance was assessed by unpaired Student t-test. *: P<0.05 between two groups. (TIF) [file pone.0181848.s005.tif]

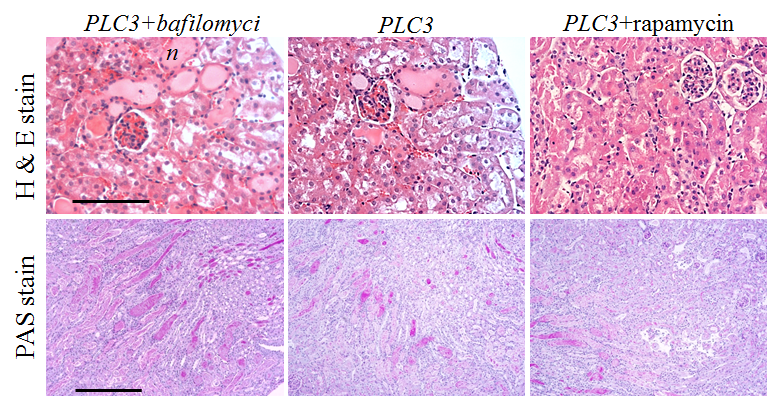

Supplement: S5 Fig — Representative H & E (upper panel, scale bar = 100 μm) stains and PAS (bottom panel, scale bar = 250 μm) stains on kidney sections of PLC3 mice pre-treated with bafilomycin A1, vehicle or rapamycin for 3 days followed by IRI for 24 hours. (TIF) [file pone.0181848.s006.tif]
